# Podcasts as a platform for sharing and disseminating experiences and expertise between young adults with cancer and radiotherapy researchers

**DOI:** 10.1186/s40900-025-00718-y

**Published:** 2025-06-17

**Authors:** Lisa Whittaker, Elena Espinosa-Cabrera, Helen Haar, Elly Hall, Sophie Lambert, Jesse Tristram, Michaela Vladykova, Rebecca Drake, Gemma Eminowicz, Patrycja Lewandowska, Catarina Veiga, Amanda Webster, Joanna McNamara, Naman Julka-Anderson, David Owen, Alice Taylor-Gee, Samantha Y. A. Terry, Jamie A. Dean

**Affiliations:** 1 Cancer Research UK RadNet City of London, London, UK; 2 School of Biomedical Engineering and Imaging Sciences, King’s College London, London, UK; 3 Patient Author, Nationwide, UK; 4 Barts Cancer Institute, Queen Mary University of London, London, UK; 5 Department of Clinical Oncology, University College London Hospitals NHS Foundation Trust, London, UK; 6 Department of Haematology, UCL Cancer Institute, University College London, London, UK; 7 Department of Medical Physics and Biomedical Engineering, University College London, London, UK; 8 National Radiotherapy Trials Quality Assurance Group, University College London Hospitals NHS Foundation Trust, London, UK; 9 Rad Chat, Sheffield, UK; 10 Gurukula Limited, Stroud, UK; 11 Institute for the Physics of Living Systems, University College London, London, UK

**Keywords:** Podcast, Young adults, Radiotherapy, Radiation oncology, Cancer research, Engagement

## Abstract

We set out to co-create a collaborative project that would raise awareness of radiotherapy, explore young adults’ experiences of cancer treatment, and share radiotherapy research with audiences beyond academia. To do this, we partnered radiotherapy researchers with young adults who had undergone radiotherapy, co-creating a series of episodes for an established oncology podcast. These conversations offered space for participants to share their stories and personal insights, both as patients and as researchers. Six young adults and six researchers took part, supported by preparatory discussions to help shape meaningful conversations. Each podcast episode featured unique patient experiences alongside different areas of radiotherapy research. An independent evaluation, based on semi-structured interviews with participants and hosts, highlighted the importance of relational connection, mutual respect, and the value of having a dedicated project coordinator. The episodes were downloaded over 3000 times in 120 countries. Participants described feeling heard and appreciated, and several researchers reflected on the renewed motivation and perspective gained through the project. This collaboration suggests how podcasting has the potential to serve as an accessible and emotionally resonant tool for engagement, enabling researchers and patients to connect in new ways and share knowledge with a broader public. Future projects of this kind may benefit from a similar co-creative and well-supported approach.

**Supplementary Information:**

The online version contains supplementary material available at 10.1186/s40900-025-00718-y.

## Abstract (plain language summary)

Patient and Public Involvement and Engagement (PPIE) in cancer research is important as it helps to align research objectives with patients’ priorities. Here, the focus was on radiotherapy, and our participants were young adults diagnosed with cancer and treated with radiotherapy, as they often have experiences of their condition and treatment unique to their age. It is important that these experiences are understood so radiotherapy is as effective as possible and patients can be given the best support and care. Researchers and young adults do not often get the chance to talk to each other. We wanted to find out whether podcasts could be used to create conversations between researchers and young people while simultaneously raising awareness of experiences and research with a wider audience. We recorded six episodes of the podcast, Rad Chat, with young adults and researchers and gathered feedback from those who took part. The podcasts were listened to worldwide. Young adults said that they felt heard and that their contributions were valued. Researchers welcomed the chance to chat with people with experience of cancer and radiotherapy and some told us this gave them more motivation for their work. We also found that the project benefitted from a dedicated project coordinator. Overall, this project showed that podcasts may be useful for engaging patients and healthcare professionals with radiotherapy research and reaching an audience beyond academia.

**Supplementary Information:**

The online version contains supplementary material available at 10.1186/s40900-025-00718-y.

## Background

In recent years, there has been a recognition and effort by policymakers, funders and researchers to involve and engage patients in research, including through the National Institute for Health and Care Research [1]. These initiatives are mainly focused on clinical research; however, involving and engaging patients in preclinical research may also be beneficial, especially when providing mutual learning opportunities to patients and researchers [2]. The perceived value of this educational opportunity has led to engagement between patients and preclinical researchers being integrated into PhD programmes [3]. While every patient’s experience is unique, there are common themes, which may vary between different patient groups. It is, therefore, important that all age groups have opportunities to engage with researchers. Many young adult patients diagnosed with cancer have different experiences of their condition and treatment to older adults and children [4]. These differences may be due to being at a time in their lives when they are gaining independence and have different psychosocial needs, different toxicity profiles [4] related to being at different stages of biological development, and their experiences with healthcare systems that are often designed around the management of children and older adults with cancer.

The most common cancers affecting young adults are thyroid cancer, germ cell tumours, lymphomas, breast cancer, colorectal cancer, central nervous systems tumours and soft tissue sarcoma [5]. Radiotherapy is a critical component of multimodality treatment for many young adults diagnosed with cancer [6]. Radiotherapy techniques indicated for treatment of young adult cancers include localised external beam radiotherapy with x-ray photons or protons, total body irradiation external beam radiotherapy as part of preparation for bone marrow transplantation and radionuclide therapies. Young adult patients commonly experience severe physical, cognitive and psychosocial side effects [7, 8], with the psychosocial side effects tending to be worse than for other age groups diagnosed with cancer [9]. Radiotherapy (and chemotherapy) contributes to chronic comorbities [10]. The effects of these cancers and their treatments often cause major disruptions to the daily lives and careers of patients [11]. Additionally, these patients have an increased risk of developing secondary cancers, including radiotherapy-induced cancers [12], compared with older adult patients.

Radiotherapy is a lesser-known treatment than surgery and chemotherapy, and patients’ experiences of it vary. Understanding these experiences is key in improving care and patient outcomes [13]. There are also misconceptions of radiotherapy held by patients, carers, the public and healthcare professionals [14]. This lack of understanding of radiotherapy may contribute to its underutilisation [15]. Enhancing knowledge levels may increase acceptance of and access to radiotherapy as an effective treatment option and improve the care and experiences of patients treated with radiotherapy. Radiotherapy research includes both clinical and preclinical research and involves a range of clinical and scientific disciplines. People with lived experience of cancer and radiotherapy are seldom able to meet non-clinical researchers and these researchers (i.e. those working in a laboratory setting with no patient contact) rarely have the opportunity to hear first-hand accounts of the experiences of people being treated with radiotherapy. To help bridge this gap, we previously designed, carried out, and evaluated a PPIE project that brought together young adults treated with radiotherapy and researchers—both preclinical and clinical—working to improve its effectiveness and reduce side effects. The aim was to share experiences, enhance understanding of radiotherapy, and develop recommendations for healthcare professionals [16]. Raising awareness of young adult cancers and the experiences of patients diagnosed with these conditions among cancer researchers may promote much-needed research to improve outcomes for this group of patients [17]. Given the perceived benefits to participants, including peer support, increased knowledge of radiation research and continued involvement in subsequent engagement projects, we reasoned that we might be able to increase the impact of our PPIE efforts by sharing our discussions between young adults treated with radiotherapy and radiotherapy researchers with a wider audience of healthcare professionals, patients and researchers.

Podcasts are an established medium for sharing stories and information with large audiences. Some podcasts focus on the narratives of young people with cancer (e.g. Not Your Grandma’s Cancer Show [18] and AfterThoughts Podcast [19]) and others highlight cancer researchers’ work (e.g. Cancer Research UK City of London Centre Podcast Series [20]). However, only a few podcasts bring young adult patients diagnosed with cancer together in conversation with researchers (e.g. Cancer Research UK That Cancer Conversation [21]). A previous project creating a podcast with young adults with cancer found that alongside the conventional methods of providing psychosocial support, it can be helpful to offer young patients with cancer other ways to express themselves [22]. The conversational nature of podcasting may also reduce the use of research jargon [23].

## What we did

In this project we aimed to increase awareness of research into improving radiotherapy for the treatment of cancer, develop researchers’ and young adults’ skills in podcasting, gain an understanding of young adults’ experiences of cancer and radiotherapy and learn from our experience to enhance future PPIE projects. We used podcasts as a medium to co-create conversations between matched radiotherapy researchers and young adults diagnosed with cancer while simultaneously raising awareness of experiences and research with a wider audience. We co-created a special podcast series through a collaboration between radiotherapy researchers, young adult patients diagnosed with cancer and treated with radiotherapy and the radiotherapy podcast Rad Chat, which provides a platform for patients to share their stories and experiences. An independent evaluator reviewed our project to highlight its strengths and identify areas for improvement, with the aim of guiding future PPIE initiatives.

### Ethics

We used the National Health Service Health Research Authority tool [24] to determine whether ethical approval was necessary. We found that the project did not constitute “research” and so ethics approval was not necessary. This decision is in agreement with recommendations for when ethics approvals should and should not be sought for PPIE activities [25] and the National Institutes of Health Research information for researchers conducting PPIE activities [26]. It should be noted that the purpose of the project evaluation was for “service evaluation” of our PPIE activity and not for “research” aiming to make generalisations to the wider population from our small number of participants. Following ethical practices, we provided participant information sheets and obtained informed consent for the podcast recording and project evaluation (see Additional Files 1–3). The contents of these documents were also discussed verbally with participants. The project evaluation responses were anonymised to everyone apart from the external evaluator (D.O.), who conducted the interviews. The recordings of the evaluation interviews were deleted within 90 days, following data security policy. All authors gave informed consent for their quotes to be included in the publication. Rad Chat offers information and support for podcast guests including briefing and debriefing, referral to support services if necessary and a complaints procedure.

The wellbeing and autonomy of all contributors were central to the design and delivery of the project. The project was coordinated by an experienced PPIE Coordinator (L.W.), who also has a background as a trained youth worker and support worker for people affected by cancer. This individual served as a consistent point of contact throughout the project, maintaining regular contact with the participants and being available to address any concerns or provide additional support if needed. The podcast content was co-created with participants and handled sensitively, with care taken to ensure contributors were comfortable with what was shared. Participants had the option to decline any topics they did not wish to discuss. The focus was on lived experience and involvement in research, rather than discussing specific clinical decisions or outcomes. The podcast hosts (J.M., N. J.-A.) are experienced, registered healthcare professionals with advanced communication training. After recording, participants were given the opportunity to review the podcast before it was published online. No material was shared publicly without their explicit consent. Participants received clear signposting to support provided by several charities and organisations that work with young people affected by cancer, including during the podcast recordings and follow-up communications.

### Recruiting young adult patients diagnosed with cancer and radiotherapy researchers

Six radiotherapy researchers were recruited through word of mouth and an advertisement in an internal newsletter. The researchers varied in their level of experience (3 PhD students, 2 early career principal investigators and 1 consultant clinical oncologist) and research topics (clinical radiation oncology, radiotherapy physics and radiation biology). Five were female and one male. For some researchers, this was their first experience of PPIE. The opportunity was presented as a chance to connect with young adults who have had radiotherapy and share their research with a non-academic audience.

Six young adult patients diagnosed with cancer were recruited to the project through charity partners: Shine Cancer Support, Trekstock and Cancer Research UK. The project was shared on social media and through invitations using contact details (previously approved for us to use for such purposes) from prior PPIE activities. We recruited a small number of participants already engaging with cancer charities. The young adults were all female and aged between 22 and 26 years. The diagnoses of the young adults included glioblastoma, Hodgkin and non-Hodgkin lymphoma, acute lymphoblastic leukaemia and germ cell cancer. They were treated with external beam radiotherapy, including total body irradiation. One participant had previously been involved in a podcast. The patient participants were reimbursed for their time.

### Preparatory discussions and matching patients and researchers

The PPIE Coordinator spoke to all young adults and researchers individually as part of the recruitment process. Initial conversations and workshops were held prior to recording the podcasts to elicit the personal stories and research perspectives that would be explored in the podcast. The young adult participants were equal partners in the planning and scripting of the podcast episodes. Three online discussions were then organised, allowing everyone to meet and discuss what they would like to get out of the podcasts and how they thought we should run the project. The young adults wanted to share their experiences of cancer and radiotherapy and learn about research, specifically research related to their diagnosis. Together, we decided that conversations between a young adult and a researcher, leading to six podcast episodes, would be the best approach to achieve these goals. Young adults and researchers were matched based on the relevance of the researchers’ work to the type of cancer that the young adult had.

### Pre-recording briefings and recording the podcasts

Podcast participant information sheets (Additional File 1) were provided to podcast participants and informed consent obtained (Additional File 2). The PPIE Coordinator facilitated a discussion and briefing with each matched pair. This was valuable as, for some pairs, it had been several months since they had previously spoken. During this briefing, young adults and researchers shared their experiences in more detail, asked each other questions and, together with the PPIE Coordinator, agreed on the focus of their podcast episode. The PPIE Coordinator drafted a script with the podcast hosts, which was shared and agreed upon with everyone in advance of the recording. The podcast hosts also chatted with each pair just before recording began for final checks that everyone was comfortable and happy to record.

Episodes were recorded remotely using Riverside.fm, with recordings lasting for around 60 minutes and were edited by the Rad Chat team. Episodes lasted between 45 and 60 minutes. The PPIE Coordinator consulted with everyone afterwards to ask how everything had gone. We released the six episodes in two blocks of three on a weekly basis. Episode descriptions and links are provided in Table 1. As of 29th July 2024, the podcast series has received approximately 3000 downloads from listeners across 120 countries.

**Table 1:** List of podcast episodes

Guests	Title and Description	Link
Jesse Tristram and Jamie Dean	Brain Tumours, Survivorship and Research - Discussion about Jesse’s cancer diagnosis, living with and beyond cancer and Jamie’s computational radiation biology research.	https://radchat.transistor.fm/episodes/bonus-episode-jesse-and-jaime-brain-tumours-survivorship-and-research
Michaela Vladyková and Patrycja Lewandowska	Hodgkin’s Lymphoma and Research - Michaela’s diagnosis, living with and beyond cancer, and Patrycja’s preclinical cancer research.	https://radchat.transistor.fm/episodes/bonus-episode
Elly Hall and Amanda Webster	Total Body Irradiation and Patient Voice in Research - Discussion about Elly’s diagnosis, living with and beyond cancer, and Amanda’s clinical radiotherapy research.	https://radchat.transistor.fm/episodes/bonus-episode-elly-and-amanda-total-body-irradiation-and-patient-voice-in-research
Helen Haar and Catarina Veiga	Non-Hodgkin’s Lymphoma - Discussion about Helen’s diagnosis, her teenage and young adult campaigning, late effects of treatment and Catarina’s radiotherapy physics research, the importance of the patient voice in research and how hearing Helen’s story has changed how she views cancer research.	https://radchat.transistor.fm/episodes/bonus-episode-helen-haar-and-catarina-correia-velosa-da-veiga-non-hodgkin-s-lymphoma
Elena Espinosa-Cabrera and Gemma Eminowicz	Germ Cell Cancer - Discussion about Elena’s diagnosis, Gemma’s career, what studying and working in medicine means.	https://radchat.transistor.fm/episodes/bonus-episode-elena-espinosa-cabrera-and-dr-gemma-eminowicz-germ-cell-cancer
Sophie Lambert and Rebecca Drake	Non-Hodgkin’s Lymphoma - Discussion about Sophie’s cancer diagnosis and late effects of treatment, and with Rebecca about her preclinical cancer research.	https://radchat.transistor.fm/episodes/bonus-episode-sophie-lambert-and-rebecca-drake-non-hodgkin-s-lymphoma

### Evaluation

After we released the first three episodes, we gathered informal feedback from a Senior Public Engagement Manager at King’s College London and the Academic Lead for Public Engagement at the Cancer Research UK RadNet City of London Centre (S.Y.A.T.). The feedback on the first three episodes was generally positive. However, some felt that, at times, the podcasts tonally resembled concurrent interviews between the podcast hosts and either the young adults or the researchers, rather than a conversation between young adults and researchers. Based on this feedback, we ran additional preparatory sessions with the final three pairs, encouraging and enabling a more conversational style in the final three episodes.

**Fig. 1:**
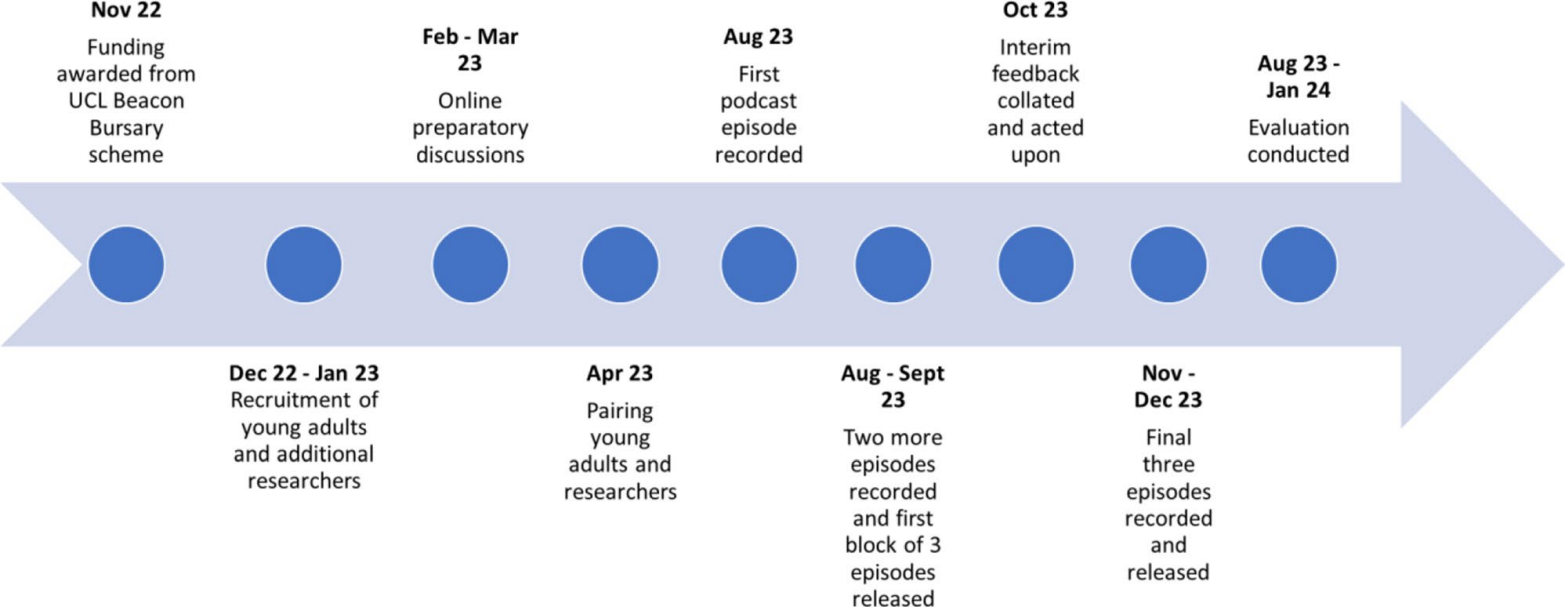
Project timeline

The project was also evaluated at completion by an independent external evaluator (D.O.), to understand the impact of the project, reveal strengths and areas for improvement and inform recommendations for future PPIE projects. It should be noted that the purpose of this evaluation was for “service evaluation” of our PPIE activity and not for “research” aiming to make generalisations to the wider population from our small number of participants. Participants were given an information sheet (Additional File 3) and informed consent obtained before agreeing to take part in semi-structured interviews to understand the experiences and outcomes of young adults and researchers. The podcast hosts and PPIE Coordinator were also interviewed as part of the evaluation. A template set of questions (Additional File 4) was followed. Follow up questions were asked to explore topics deeper or clarify answers. Where a strong theme had emerged from other interviewees, this was tested towards the end of the interview to further deconstruct or validate the emerging theme. During the semi-structured interviews, notes were taken and an initial set of themes was identified. The interviews were then transcribed using Otter.ai, transcripts were reviewed and a set of reporting themes was identified. This was performed using Microsoft Word due to the low number of interviewees. Sections of the transcript were corrected by the external evaluator where needed. An evaluation report was produced based on these interviews (Additional File 5). This evaluation report was reviewed and discussed collaboratively by the co-authors (including the young adult participants and researchers), and together we agreed on which quotes to include in the manuscript to best illustrate key points and shared reflections. A timeline for the complete project is shown in Fig. 1.

## What we learned

As with our previous PPIE projects [16], flexibility was required in coordinating and running this project. We originally planned to record four podcast episodes, but our initial discussions revealed that having six pairs recording episodes would be most interesting and valuable, so we adjusted our timescales allowing more time for the project to be completed and two additional episodes to be recorded. We learned that young adults were not motivated by any financial reimbursement for their time, as this was not mentioned until they were recruited.

### Young adult participant feedback

**Fig. 2:**
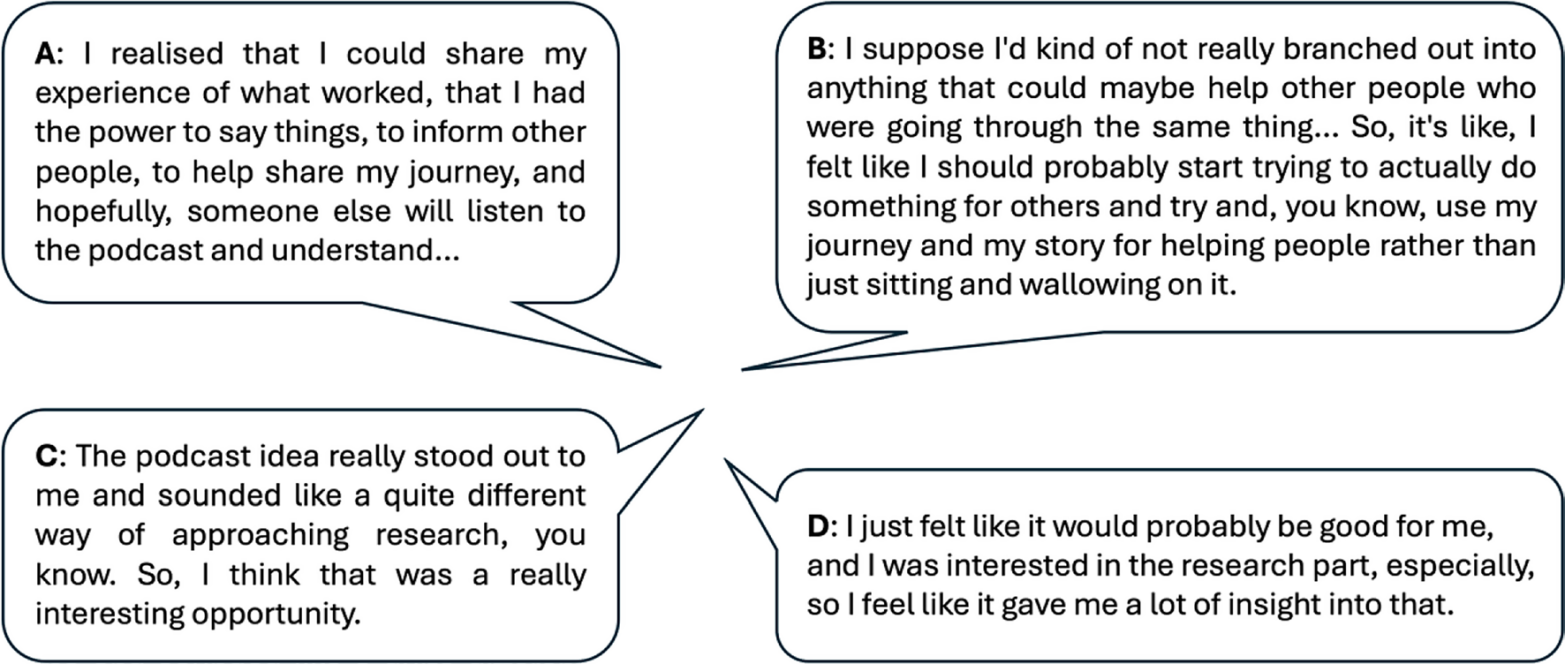
Motives of young adult participants for taking part

Overall, we learned that our podcast series provided a meaningful platform for young adults with experience of cancer and radiotherapy to share their experiences with peers, researchers, professionals and the wider public. Young adults’ motives for taking part varied, although three main themes emerged: (i) to help other people who may be experiencing similar challenges (Fig. 2A); (ii) to act as a role model for others with their condition and a moral obligation to bring a positive story into the public forum (Fig. 2B); and (iii) to find out more about research being undertaken on their condition and to meet professionals who are working in this area (Fig. 2C and D).

**Fig. 3:**
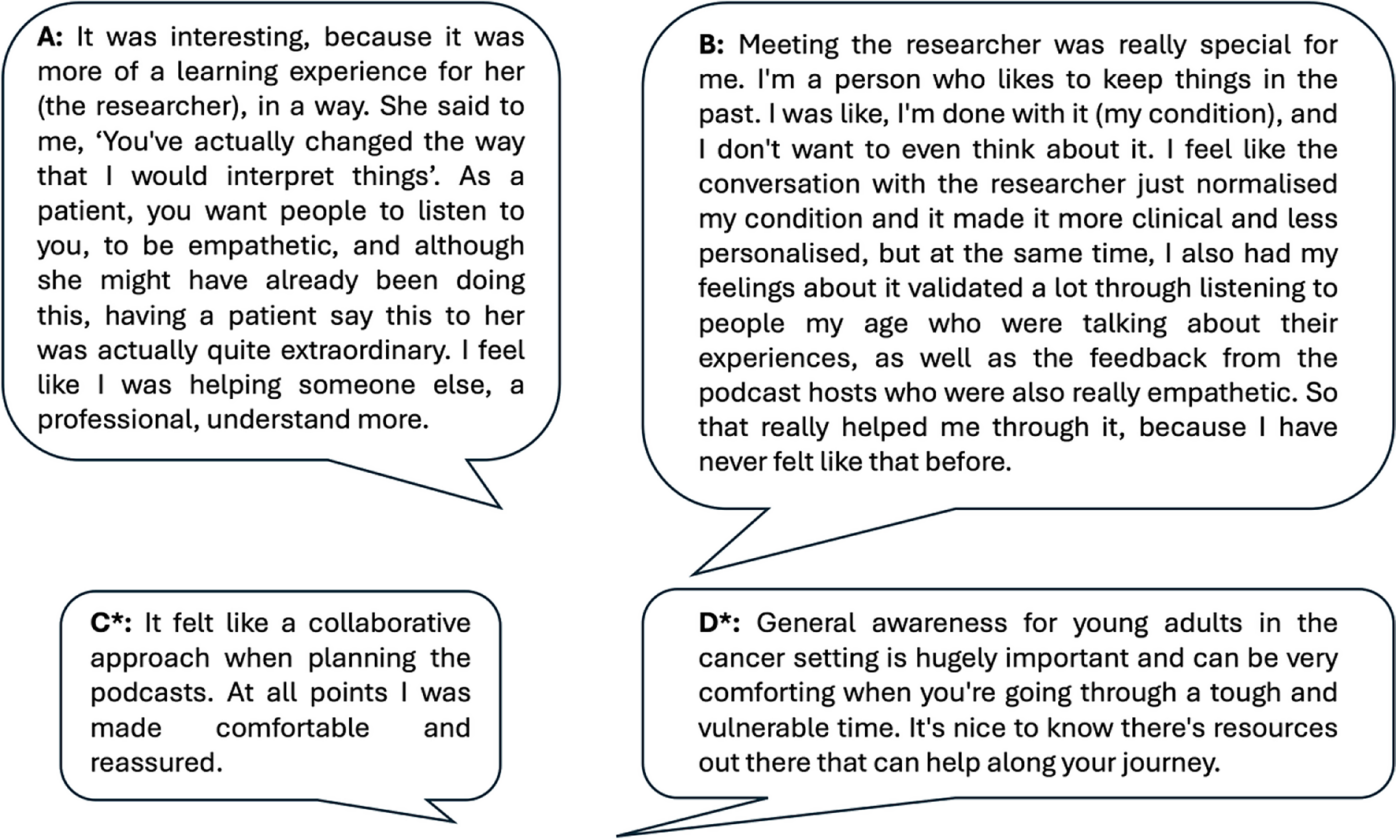
Feedback from young adult participants. *Quote from young adult participant in correspondence subsequent to the independent project evaluation

Young adults valued the opportunity to meet with researchers and other young adults with cancer at the outset of the project (Fig. 3A). The preparatory discussions were considered a good way to ease into the project and find out more. For one young adult, the link with research provided a helpful focus for conversations and was a key reason for them taking part. They stated that it offered a different experience and level of conversation, reducing the emotional burden and vulnerability compared to talking with patients with a similar condition to them (Fig. 3B). The young adult participants felt that they were involved as partners in the project rather than merely consulted (Fig. 3C). They expressed the importance, from their perspective, of raising awareness and providing resources that could help other young adults navigating similar experiences (Fig. 3D).

**Fig. 4:**
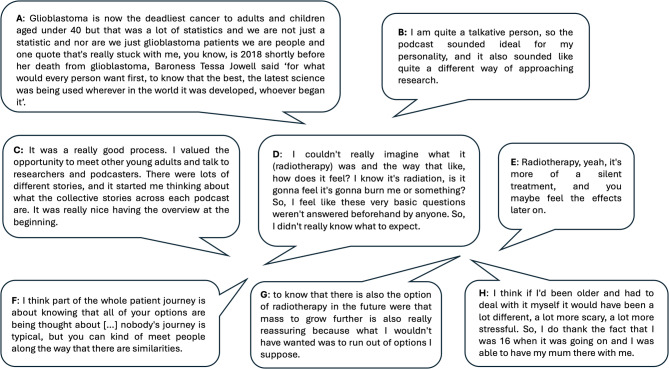
Excerpts from the podcast episodes and evaluation from young adult participants

Some of the participants appeared to take on the messages that researchers brought to the podcast (e.g. the lack of funding for research, that they could contact researchers directly) and, therefore, may potentially advocate for more research in the future (Fig. 4A). The platform of podcasting appeared to suit many participants (Fig. 4B). The project was described as a good opportunity to meet other young adults, talk to researchers and podcast hosts, and listen to and share different stories and experiences (Fig. 4C). Our external evaluator concluded that overall, young adult participants felt heard and welcomed as part of the process and that their partnership and contributions were valued. It should be noted that all young adult participants co-authored this manuscript, contributing directly to the writing and editing process. The young adults’ interactions with researchers were viewed as rewarding, due to acquiring new knowledge about the research process and the people researching their condition. Specifically, they spoke about not being seen as a statistic and the need for research into their specific type of cancer (Fig. 4A), having a lack of knowledge and information about radiotherapy (Fig. 4D and E), the importance of having options and knowing all of these are being considered (Fig. 4F and G), the unique position of being a young adult and the importance of family support (Fig. 4H).

### Researcher participant feedback

**Fig. 5:**
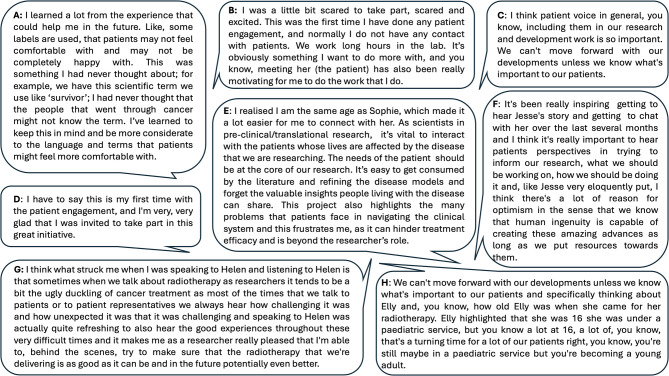
Excerpts from the podcast episodes and evaluation from researchers

The project was generally well received by researchers. The time commitments were manageable, and there were clear benefits for the researchers who took part. The project provided an opportunity to engage with people directly affected by their research; for some non-clinical researchers, this was a rare opportunity and acted as a motivating factor, bringing more meaning to their jobs (Fig. 5A and B). Overall, researchers found the interactions with young adults rewarding as they provided meaning and a human context to their work. Specifically, they spoke about the importance of PPIE (Fig. 5C). For several of the researchers, this was the first time they had spoken to a patient and, they found the experience valuable (Fig. 5D); many of the researchers involved connected with the young adults (Fig. 5E and F) and met up in person for lab tours. One young adult spoke about her radiotherapy treatment positively, which was surprising and encouraging for the researcher to hear (Fig. 5G) as was recognition of the experience of young adults often transitioning between a paediatric and adult service (Fig. 5H).

**Fig. 6:**
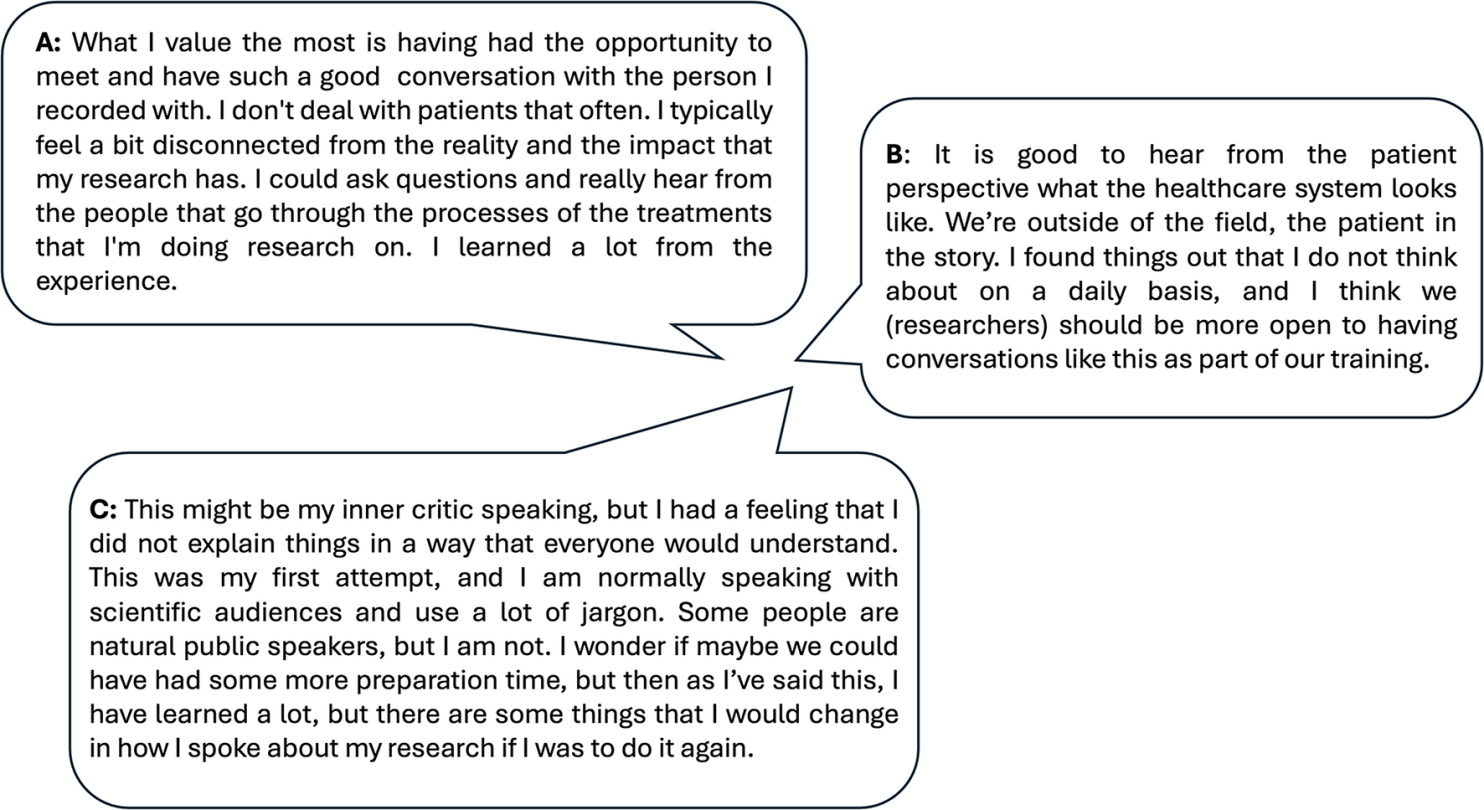
Feedback from researchers

Additionally, researchers found the experience educational, through providing an opportunity to practice effectively communicating their research. The programme was considered suitable for researchers with both little and copious previous experience of PPIE (Fig. 6A and B).

### Need for a dedicated PPIE coordinator

The facilitatory role of the PPIE Coordinator was seen as essential in bringing together the project’s different components and helping to develop the storyline. They provided the necessary project coordination and facilitation to help manage the process and ensured participation was beneficial and impactful to all involved. Given the potential challenges in matching participants with researchers, the fact that all participants felt well-matched, and the podcast conversations were coherent, this can be viewed as a key success in the delivery of the project. Several participants referred to the Coordinator’s support as vital in helping build their confidence to participate in the podcast and help prioritise which parts of their story they would share in the podcast.

### Beyond the podcast series

We found that once rapport had been formed between young adults and researchers, some continued to be in contact following their podcast recordings. This relational aspect of the project was also valued by participants. One young adult reported that the researcher they were matched with had subsequently invited them to their lab, and another said they had been in touch after the project to keep up conversations about research. Another reported that they valued the opportunity to bring some meaning to the work that the researchers are doing. A further young adult discussed that their conversations with the researcher helped alter their relationship with their condition. Six participants took in a subsequent PPIE activity on using art as a medium to explore what radiotherapy means and looks like from their perspective. The PPIE Coordinator and one of the young adults gave a joint presentation about the project at the UK Imaging and Oncology Congress. Two participants who were matched together for a podcast episode were involved in a television interview to raise awareness of the cancer that they were diagnosed with/researching and a television advertisement to raise funding for cancer research. It was clear that much was gained from the project in addition to the six podcast episodes we recorded. This further emphasises the value of conducting PPIE activities.

### Areas for improvement

We note that our cohort of young adult participants is not representative of the overall population of young adult patients diagnosed with cancer. Ideally, we would always aim to recruit a diverse group of participants and try to reach those who may not normally take part in PPIE projects. We shared the opportunity widely and only needed to recruit a small number of participants. Our aim for diversity had to be balanced with not wanting to turn away those who got in touch wishing to take part as it might put them off future PPIE activities. We acknowledge that a different or more targeted recruitment process may increase diversity in future projects.

As our participants were new to podcasting, we tried to prepare them. However, we learned from our evaluation that we could have done more to ensure participants felt fully prepared and comfortable ahead of the recording (Fig. 6C). We therefore recommend that others undertaking similar projects with podcasts consider a short preparation session or rehearsal, particularly for researchers or for patients and researchers together to build confidence. As part of this session, both researchers and patients could be supported with some training, e.g., in how to answer difficult or surprising questions, how to engage in personal and potentially emotional conversations, managing your boundaries and authenticity.

The evaluation suggested that the level of support for participants was considered appropriate. Potentially, there could be an opportunity for a rehearsal or role-play in the early part of the podcast to help build confidence; equally, the programme team might consider a debrief with researchers to reflect on the experience and lessons learned. This debrief could be facilitated with all participating researchers and would invite them to reflect on what they have learned about PPIE, and how they might apply what they have learned moving forward.

Several of the patient participants had already participated in previous patient engagement, involvement or advocacy work and were used to talking about their experiences. However, the format of podcasting (as a form of recorded media) did create some challenges, and it is worth considering light-touch ‘media training’ for both participants and researchers or the chance to edit out parts of the episodes.

## Conclusion

In conclusion, the project delivered its stated aims, by providing a successful format for engaging researchers and young adults with experience of cancer in conversations and gaining an understanding of their experiences and sharing these conversations with a wider audience. Engaging patients diagnosed with cancer with research related to their disease was deemed to be a valuable experience. It enabled researchers to practice communicating their research to non-specialist audiences. Researchers gained some familiarity with the format of podcasts as a mechanism to communicate research and were left feeling positive about PPIE. While it was not a stated aim, the fact that the experience also provided a much-needed channel for patients diagnosed with cancer to share their experiences and potentially inspire other patients diagnosed with cancer and professionals contributed to the success of the programme and was key to its mutual benefit. We recommend that researchers and healthcare professionals consider co-creation of podcasts with individuals who have lived experience of treatment as a potential design for PPIE projects. This approach, when supported by a dedicated PPIE professional, may build connections, amplify patient voices, and extend the reach of research.

## Electronic supplementary material

Below is the link to the electronic supplementary material.

Supplementary Material 1: Additional File 1. Title of data: Rad Chat podcast information sheet. Description of data: Information sheet for participation in the Rad Chat podcast.

Supplementary Material 2: Additional File 2. Title of data: Rad Chat podcast consent form. Consent form for participation in the Rad Chat podcast and the use of related materials for communication, promotion and education.

Supplementary Material 3: Additional File 3. Project participant evaluation information sheet. Description of data: Participant information sheet given to participants before they were interviewed as part of the evaluation of the project.

Supplementary Material 4: Additional File 4. Title of data: External evaluation interview questions. Description of data: Questions used in the semi-structured interviews of participants by the external evaluator.

Supplementary Material 5: Additional File 5. Title of data: External evaluation report. Description of data: External evaluation report summarising the participant interviews and podcast audience data.

## Data Availability

Hyperlinks to access the podcast episodes are provided in Table 1. Participant information sheets and consent forms and the independent evaluation interview questions and report produced by D.O. are included as Additional Files.

## Abbreviations

PPIE: Patient and public involvement and engagement
