# Supplementary material for: Podcasts as a platform for sharing and disseminating experiences and expertise between young adults with cancer and radiotherapy researchers
Source: Res Involv Engagem. 2025 Jun 17;11:64. doi: 10.1186/s40900-025-00718-y (PMC12172223; doi:10.1186/s40900-025-00718-y)
Supplement: Supplementary file 1 — Supplementary Material 1: Additional File 1. Title of data: Rad Chat podcast information sheet. Description of data: Information sheet for participation in the Rad Chat podcast. [file 40900_2025_718_MOESM1_ESM.pdf]

RadChat Information and support for guests

Welcome to to your Rad Chat podcast episode guide! **Don't know about us or the podcast? Check out our bio's**.

Thank you for agreeing to take part in educating and supporting others through our podcast. We're proudly and passionately committed to making the world a better place through radiotherapy and oncology education. Your episode will be broadcast to over 122+ countries and our 10K+ followers worldwide as free continual professional development (CPD).

After publishing over 300 episodes, we have collated some frequently asked questions below to help you feel prepared for a chat with us.

FAQs

Where and how is the episode recorded?
We record in a virtual studio (similar to teams/zoom) called Riverside FM, a link will be sent to your email. You must open this link in google chrome on a laptop or PC only.

Do I need a microphone for the recording?
If you have an external microphone please use it, but you do not need to go out of your way to purchase one for this episode. If you don't, using a microphone built into headphones is okay too. If you don't have either, we can use your computer's in-built microphone but the sound quality is lower.
If you have a microphone attached to headphones just be conscious that it doesn't rub on material whilst talking.

Do I need headphones for the recording?
Wearing headphones makes your audio 'cleaner' as your microphone will not accidently pick up the voices of other people on the call.

Can I do a test run to see if things work?
Yes, we recommend you test the Riverside FM link before our recording along with connecting your camera and mic.
You may also need to check your browser settings, because if you have your default browser set as something else you will need to copy and paste the link into google chrome.

Is the recording being live broadcasted?
No, your episode is pre-recorded and will be released at a later date. We edit all the episodes ourselves and there will be an opportunity to listen to the final version prior to release should you wish to.

Do you record me on camera?
Yes, but we only use small 30-60second snippets from the episode that we edit into a clip to advertise your episode on social media. If we intend to video the entire episode for our You Tube channel you will be informed.

What are you going to ask me?
Our podcast is relaxed, authentic and a 'chat' where we avoid the conversation being too scripted. We will only ever ask you questions related to your personal and professional experiences. When you scroll further down, you'll see topics we'd like to cover and our structure. We may add points/stats, or examples/stories we want to bring out in the conversation in addition to the questions we ask you. Ultimately, this is your time to shine! We're here to get the best out of you for our listeners.
If you have a particular question you'd like us to ask you then feel free to add it to the template below.

Will you ask me anything sensitive?
We are registered healthcare professionals with experience of working with people living with and beyond cancer and both have advanced communication training. We are not journalists looking to elicit an emotional or negative response from you, nor are we looking for click bait. If we ask you about your personal experience, we will always do so sensitively and carefully. Also, if there is something you do not want to answer, there is no obligation to do so! We always check in with our guests afterwards. Your wellbeing is always important to us and if at anytime you don't feel comfortable or want to stop it is absolutely fine to do so.

Can I suggest topic(s) to cover?
Absolutely, this is a dynamic document which automatically saves when you edit it. when you scroll down to the topics / questions, please feel free to add to it.

What happens if I make a mistake or swear during the episode?
No worries, during the recording you can pause and restart. All our guest episodes are edited with music and only released in the best quality. We want to show you off and wouldn't release something that portrayed you (or us) in a negative light.

Can I listen to the episode before it goes live?
Absolutely, once we have edited the episode to a release quality, we can share a link with you.

What if I listen back and want some edits made?
We are happy to edit sections where you feel there is a need. Our podcast is relaxed, authentic and a 'chat' meaning we'd like to avoid re-recording, or big edits.

Does my workplace / employer need to check this?
We will leave this to you, if you need us to send a link to share your episode please let us know asap. Ideally we would like this to happen asap to ensure we release your episode in a timely manner.

Does anyone else check the episode?
All our episodes are checked by at least two people from the Rad Chat team (registered healthcare professionals) to ensure sound quality, fact check and provide us with feedback.

What do you need from me?
Phew, you're still here which means you are nearly ready for the lime light…

Before we can record, we need a bit of information from you please. (This is a dynamic document which automatically saves).

- Full name:
- Phonetic pronunciation of your name:
- What you like to be called:
- Pronouns:
- Mobile number (in case of any technical difficulties):
- Best email to contact you on:
- Social media accounts we can tag you on:
- Next of kin or emergency contact (only to be used if there is a problem during the recording):
- Short bio:
- Links to news articles, journal articles, research, literature etc you want us to put in the show notes:
- Please email us a photo of you we can use for social media to promote this episode on rad_chat@outlook.com

If you have any further questions, concerns or anything whatsoever just get back in touch with us asap please either by email, phone or social media.

By sending us any of the above information, photos and sharing your story you are consenting with us to share this across the world across all of our platforms.

Please also note that by sending us your social media details you are consenting to us tagging you in posts. Please refer to the consent form we have sent you. If at any point you want to withdraw consent please let us know.

All the content we create is owned and copyrighted by Rad Chat.

Episode guide and structure:

Intro (us):
Hello everyone and welcome to the Rad Chat, founded by me Jo McNamara and me Naman Julka-Anderson.

Rad Chat is a forward-thinking global knowledge hub where healthcare professionals can advance their expertise in therapeutic radiotherapy and oncology. Unlike traditional academic resources, we blend real-world experience, expert insights, best practice and patient perspectives.

We make advanced knowledge engaging and accessible, supporting continuous learning and professional development without compromising patient care or personal time. By providing insights into both technical skills and career development, helping you progress confidently in your field and shape your professional future.

Just to let you know, our episodes may contain sensitive and difficult topics that you might distressing or triggering. Please consider checking out another episode.
