# Supplementary material for: Podcasts as a platform for sharing and disseminating experiences and expertise between young adults with cancer and radiotherapy researchers
Source: Res Involv Engagem. 2025 Jun 17;11:64. doi: 10.1186/s40900-025-00718-y (PMC12172223; doi:10.1186/s40900-025-00718-y)
Supplement: Supplementary file 2 — Supplementary Material 2: Additional File 2. Title of data: Rad Chat podcast consent form. Consent form for participation in the Rad Chat podcast and the use of related materials for communication, promotion and education. [file 40900_2025_718_MOESM2_ESM.pdf]

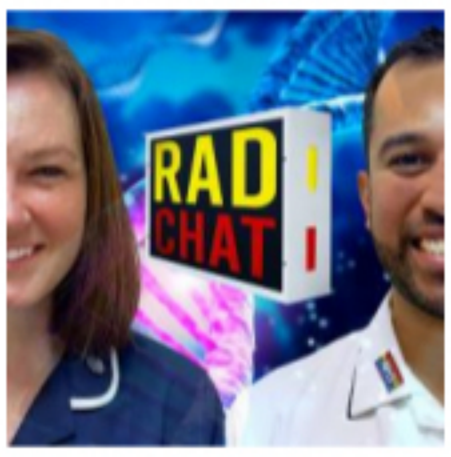

CONSENT FORM

Rad Chat Limited produces a range of communications and resources to help support people navigating a cancer diagnosis, treatment and beyond. The materials aim to raise awareness, share important messages and empower people to help others.

We would use your photography, footage, audio clips and story in our communications, educational resources and social media. By completing this form, you are giving us permission to use your content. If we receive external requests to access our content or collaborate, you will be notified to ensure you consent to this and be credited if you wish to be. We will try to keep all patient information anonymised and care will be taken to exclude any distinguishing features e.g. identifying tattoos, facial features etc.

You are entitled to withdraw your consent for any future use of your personal information and images at any time - please contact us via email radchat@outlook.com with your full details.

I am happy to give my permission

Please sign this form to show you have read the Rad Chat Consent Terms and Conditions (Link below) and are happy to give permission for your photography, footage and story to be used by Rad Chat for the purposes outlined above.

Full name

Signature Date

Please read our privacy policy below for more information on how we process your data: Rad Chat Consent Terms and Conditions

Rad Chat is a registered business in England and Wales (No. 14553489).
