# Supplementary material for: Podcasts as a platform for sharing and disseminating experiences and expertise between young adults with cancer and radiotherapy researchers
Source: Res Involv Engagem. 2025 Jun 17;11:64. doi: 10.1186/s40900-025-00718-y (PMC12172223; doi:10.1186/s40900-025-00718-y)
Supplement: Supplementary file 3 — Supplementary Material 3: Additional File 3. Project participant evaluation information sheet. Description of data: Participant information sheet given to participants before they were interviewed as part of the evaluation of the project. [file 40900_2025_718_MOESM3_ESM.pdf]

Participant Information Sheet

Project Title: Special Podcast Series

You are invited to take part in this evaluation project. Before you decide to do so, we invite you to understand why the evaluation is being done and what it will involve. Please take time to read the following information carefully and discuss it with others if you wish. Ask us if there is anything that is not clear or if you would like more information.

Aims of the Project?

We'd like to learn more about your experiences participating in the Special Podcast Series. Our aim is to find out more about what you enjoyed, learned or valued about taking part and any recommendations for future projects of this nature. You'll be invited to a short 30min interview with David Owen (an external evaluator). These interviews will help inform a short report for University College London to help them assess the impact of the work and develop future projects of this kind.

Why have you been chosen?

We'd like to talk to a range of people who have been involved in the podcast project. If you decide to participate, you can keep a copy of this information sheet, and you should indicate your agreement to the consent form. You can still withdraw at any time. You do not have to give a reason.

Will my taking part in this project be kept confidential?

All information we collect about you during the evaluation will be kept strictly confidential. You cannot be identified or identifiable in any reports or publications. If any material is reported that would make you personally identifiable, we will make every effort to seek your consent before sharing this material with University College London

Will I be recorded, and how will the recorded media be used?

The interviews will be recorded to help with analysis but will only be used by the evaluation team.

Any information you provide will be treated as confidential to Gurukula Limited and stored securely. Without your consent, we will not use your name or job title in any reporting.
Please see our privacy policy for more information about how we collect, store and process research data

What type of information will be sought from me, and why is the collection of this information relevant for achieving the research project's objectives?

We're interested in exploring what participating in the Podcast project means to you. To help us do this, we will be asking a range of questions that explore your:

- Motivations for getting involved, for example, how you first heard about the Podcast project? What motivated you to take part?
- Your experiences of taking part, for example: Did you learn something new? What did you value about the experience? What could have been different?
- Do you have any recommendations about how we can improve future episodes of the podcast?

Please contact [redacted] or by telephone at [redacted] if you have any further questions about t is wor .
