# Supplementary material for: Podcasts as a platform for sharing and disseminating experiences and expertise between young adults with cancer and radiotherapy researchers
Source: Res Involv Engagem. 2025 Jun 17;11:64. doi: 10.1186/s40900-025-00718-y (PMC12172223; doi:10.1186/s40900-025-00718-y)
Supplement: Supplementary file 4 — Supplementary Material 4: Additional File 4. Title of data: External evaluation interview questions. Description of data: Questions used in the semi-structured interviews of participants by the external evaluator. [file 40900_2025_718_MOESM4_ESM.pdf]

Topic Guide

Project Title: Special Podcast Series

Provide a short introduction to the evaluation including consent.

Key interview questions for participants

- Can you share briefly how you were involved in the podcast? (e.g., what role, what you did etc.?)
- Please tell us a bit more about how you heard about this podcast and why you got involved?
- Do you listen to podcasts usually? Any sort?
- What did you most value about taking part?
- Did you feel comfortable taking part? What was it like talking about your diagnosis/research?
- Did you learn anything new? (e.g., explore what the researcher learned from the participant and vice versa, learning from the process/experience, learning through reflection etc.)
- Has anything changed as a result of taking part in this project?
- Can you tell us more about the experience of talking with the other participant? Did you learn something from each other? What are your takeaway memories?
- What do you hope the impact of the podcast will be on its listeners?
- Do you feel podcasts like this are an effective way to share your research/story?
- Is there anything you would change about the experience?
- Did you feel well-supported in the role?
- How was the communication and management side of things?
- Have you shared the podcast with anyone? What did they say?
- Is there anything that I have not asked you that you would like to explore?
