# Supplementary material for: Podcasts as a platform for sharing and disseminating experiences and expertise between young adults with cancer and radiotherapy researchers
Source: Res Involv Engagem. 2025 Jun 17;11:64. doi: 10.1186/s40900-025-00718-y (PMC12172223; doi:10.1186/s40900-025-00718-y)
Supplement: Supplementary file 5 — Supplementary Material 5: Additional File 5. Title of data: External evaluation report. Description of data: External evaluation report summarising the participant interviews and podcast audience data. [file 40900_2025_718_MOESM5_ESM.pdf]

DavidOwen
Consultant | Researcher | Facilitator

RadChat Special Podcast Series

Summary Evaluation Report

David Owen

Introduction

This special podcast series was a collaboration between researchers funded by CRUK RadNet City of London, young adults with experience of cancer and radiotherapy and the award winning podcast RadChat. The project was funded by a Beacon Bursary from University College London. The project brought together six teenagers and young adults (TYAs) diagnosed with cancer, with six radiation researchers to co-produce a special series of podcasts The project was co-ordinated and facilitated by a Patient and Public Engagement (PPIE) Co-ordinator,

TYAs were matched with researchers working in research areas that directly impacted the lives of cancer patients. Initial conversations and workshops were held prior to recording the podcast in order to elicit the personal stories and research angles that would be explored in the podcast.

The aims of the project were to:

- Increase awareness of radiotherapy as an anti-cancer treatment.
- Improve researchers' and TYA's skills in podcasting.
- Gain an understanding of TYA's experience of cancer.
- Raise knowledge of impactful Patient/Public Involvement and Engagement (PPIE).
- Promote UCL's cancer research to new audiences outside of the academic domain.

This report aims to provide a summary of the experiences of participants taking part in the project, lessons learned, and the outcomes arising.

The findings and recommendations detailed here are informed by:

- Semi-structured interviews with a selection of participants (4 x researchers; 4 x TYAs)
- Audience survey (3 responses)
- Semi-structured interview with RadChat producers and hosts (2 producers)
- Ongoing discussions with the PPIE Co-ordinator (1 coordinator)

The recorded outputs from the programme are included in the final section.

Summary of Key Findings

In the following section, we have brought together the key findings from our evaluation. This is broken down into the following sections:

- Experiences and outcomes for patients.
- Experiences and outcomes for researchers.
- Commentary and lessons learned from the process.
- Insights from listeners.
- Conclusions and recommendations.

Experiences and outcomes for patients

- Some participants had had previous experience of patient involvement, and talking about their condition with peers, specialists or public audiences; for others, this project was their first experience.
- Participants' motives for taking part were varied; however, three main themes emerged from discussions: (i) To help other people who may be experiencing similar challenges; (ii) to act as a role model for others with their condition and a moral obligation to bring a positive story into the public forum; and (iii) to find out more about research being undertaken on their condition and to meet professionals who are working in this area.

> *I just felt like it would probably be good for me, and I was interested in the research part, especially, so I feel like it gave me a lot of insight into that…*
>
> *I suppose I'd kind of not really branched out into anything that could maybe help other people who were going through the same thing… So, it's like, I felt like I should probably start trying to actually do something for others and try and, you know, use my journey and my story for helping people rather than just sitting and wallowing on it…*
>
> *The podcast idea really stood out to me and sounded like a quite different way of approaching research, you know. So, I think that was a really interesting opportunity…*
>
> *I realised that I could share my experience of what worked, that I had the power to say things, to inform other people, to help share my journey, and hopefully, someone else will listen to the podcast and understand…*

- Participants valued the opportunity to meet with researchers and other cancer patients at the outset of the project. This was viewed as a good way to ease into the project and find out more.
- For one participant, the link with research provided a helpful focus for conversations and was a key reason for them taking part. They felt it offered a different experience, and level of conversation, reducing the emotional burden and vulnerability of just working with patients with a similar condition as they had.
- The 'human' or relational aspect of the programme was also valued. One patient reported that their researcher match had subsequently invited them to their lab, and

another said they had been in touch after the project to keep up conversations; another reported that they valued the opportunity to bring some meaning to the work that the researchers are doing; another discussed that their conversations with the researcher helped alter their relationship with their condition.

> *Meeting the researcher was really special for me. I'm a person who likes to keep things in the past. I was like, I'm done with it (my condition), and I don't want to even think about it. I feel like the conversation with the researcher just normalised my condition and it made it more clinical and less personalised, but at the same time, I also had my feelings about it validated a lot through listening to people my age who were talking about their experiences, as well as the feedback from the podcast hosts who were also really empathetic. So that really helped me through it, because I have never felt like that before.*

> *It was interesting, because it was more of a learning experience for her (the researcher), in a way. She said to me, 'You've actually changed the way that I would interpret things'. As a patient, you want people to listen to you, to be empathetic, and although she might have already been doing this, having a patient say this to her was actually quite extraordinary. I feel like I was helping someone else, a professional, understand more.*

- Some of the participants appeared to take on the messages that researchers brought to the podcast (e.g. the lack of funding for research, that they could contact researchers directly) and, therefore, may also become advocates for more research in future activities.

> *I didn't consider that even patients, and people who are having struggles with treatment, or making sense of articles, can contact the researchers directly and ask about questions about it. Not everyone reads scientific papers, but some people who have rare cases of cancer or rare treatments might be looking for answers, so that is something I never thought of.*

- The programme overall was well received as a good opportunity to meet other young adults, as well as talk to other researchers and the podcast team, and listen to and share different stories and experiences. The platform of podcasting appeared to suit many participants.

> *It was a really good process. I valued the opportunity to meet other young adults and talk to researchers and podcasters. There were lots of different stories, and it started me thinking about what the collective stories across each podcast are. It was really nice having the overview at the beginning.*

> *The researcher I connected with was so lovely. We have been in touch since we recorded the podcast. It really helped that they knew immediately just how awful (my condition) is; being with someone who knows how bad it is but is also who cares deeply about trying to improve it was actually quite special.*

> *I am quite a talkative person, so the podcast sounded ideal for my personality, and it also sounded like quite a different way of approaching research.*

Experiences and outcomes for researchers

- For some researchers, this was their first experience of PPIE; for others, it was their first experience of PPIE with patients or using a podcast as a platform; for others, this built on previous relatable experiences. The programme successfully accommodated this range of experience and provided sufficient support for all participants, although researchers for whom this was their first experience suggested areas where additional support would be welcome (this is covered in the next section).
- The programme provided an opportunity to engage with people directly affected by their research; for some researchers, this was a rare opportunity and acted as a motivating factor, bringing more meaning to the day job.

> *I was a little bit scared to take part, scared and excited. This was the first time I have done any patient engagement, and normally I do not have any contact with patients. We work long hours in the lab. It's obviously something I want to do more with, and you know, meeting her (the patient) has also been really motivating for me to do the work that I do.*
>
> *What I value the most is having had the opportunity to meet and have such a good conversation with the person I recorded with. I don't deal with patients that often. I typically feel a bit disconnected from the reality and the impact that my research has. I could ask questions and really hear from the people that go through the processes of the treatments that I'm doing research on, I learned a lot from the experience.*
>
> *Especially when working on a disease where the outcomes are not positive, I often ask myself, 'Why am I spending my career working on this?'. It is nice to have had an experience to counterbalance this.*

- The evidence suggested that this was a positive experience of PPIE for all researchers and would lead to further or continued engagement.
- Some of the researchers noted that the patients were very confident with talking about their experiences, and that this helped to keep a balance between researchers and patients in the podcasts.
- It was evident that the programme was a clear professional development opportunity for researchers, building their skills and experience in patient involvement and also communicating their research to non-specialist audiences. One of the early career research participants suggested that there could be an optional debrief about the programme to help them reflect on their experiences and build confidence for future engagement opportunities.

> *This might be my inner critic speaking, but I had a feeling that I did not explain things in a way that everyone would understand. This was my first attempt, and I am normally speaking with scientific audiences and use a lot of jargon. Some people are natural public speakers, but I am not, I wonder if maybe we could have had some more preparation time, but then as I've said this, I have learned a lot, but there are some things that I would change in how I spoke about my research if I was to do it again.*

- The programme overall was positively received by researchers, the time commitments were manageable, and there were clear benefits for the researchers who took part, including:
    - The opportunity to engage with patients and hear their experiences was viewed as a motivator for continued research but also as a learning experience.
    - Building confidence in patient and public involvement, including developing ways to talk about research with non-specialist audiences and communicating with empathy.
    - The chance to raise the profile of their research with a broader public and professional audience.

> *I learned a lot from the experience that could help me in the future. Like, some labels are used, that patients may not feel comfortable with and may not be completely happy with. This was something I had never thought about; for example, we have this scientific term we use like 'survivor'; I had never thought that the people that went through cancer might not know the term. I've learned to keep this in mind and be more considerate to the language and terms that patients might feel more comfortable with.*

> *It is good to hear from the patient perspective what the healthcare system looks like. We're outside of the field, the patient in the story. I found things out that I do not think about on a daily basis, and I think we (researchers) should be more open to having conversations like this as part of our training.*

> *The young person we worked with highlighted that she just wanted to be treated as a human. She just asked me how I am, how my day is, there is no special training in all this, you just need to take a pause, it is not rocket science, but sometimes they are just after human connection. I came out thinking, we just have to be human with each other, at the end of the day.*

Commentary and lessons learned from the process

- The Public and Patient Engagement Coordinator role was viewed as being vital to the process. This person provided the necessary project coordination and facilitation to help manage the process but also ensured that participation was beneficial and impactful to all those involved. Several participants pointed to the support of the coordinator as being vital in helping build their confidence to participate in the podcast and help prioritise which parts of their story they would share in the podcast.
- Participants felt well-matched, and the matching process was thought to be very successful.

> *I could ask questions and really hear from the people that go through the processes of the treatments that I'm doing research on.*

> *The aim of the goal of these episodes was to raise awareness and also to connect the patient with the researchers. It was really nice to work with everyone involved*

and to connect with people who can give you another insight into your work. A key part of the project was engagement with other professionals (those listening to the podcast), and I hope to do more of this in the future.

- Researchers suggested that it would have been good to do more with the podcast producers beforehand. This could be to build their confidence. One researcher suggested they would have liked the option for a dress rehearsal, given this was their first experience. The podcast team supported the observations of researchers and recognised that some of the researchers participating could have benefited from further support for building their confidence.

  We could have done a bit more prep, perhaps a mockup session. The first five to ten minutes were hard. A small dress rehearsal would have taken the edge off it.

  We could have done more preparation. The patients seemed to feel at ease, but the researchers struggled a little more to relax into it.

  I think it was fine. Because that I had done one before. And the time I did it before, I was just thrown into it, but at least this time, we had some opportunity to meet people beforehand. But at the same time, there's a part of me that thinks maybe we could have done a little bit more preparation, I think it would have been really useful maybe to have had, even if it was as a bigger group, a bit of a mockup session.

- Some participants wondered if the next step was to develop a more integrated storyline. People reflected that the podcast sometimes felt like two separate interviews, with the patients going first. People acknowledged that to do this differently, there would have to be a great deal more time invested in researching and producing the podcast season. Overall, participants felt that the podcast conversations were natural and flowed well.

  The conversation was natural for me. But it was quite separated, so for example, it was more like my side of the story and then the researcher's side of the story, although it did become a bit mixed later.

  The conversation flowed really well, also it wasn't live, so I felt kinda calm about the fact that they could edit it out. But later, they told us they don't really edit it out. So yeah, like the awkward pauses and everything. It all stayed there. And I was like, oh, yeah, but it was good.

  It's quite hard actually to marry the two stories. There's research and there's this, this person that has a patient experience, and how do you get them together? I think it was a bit easier for me because I read some of my patient's research before we met so we could discuss this on the podcast. It feels important to find things to connect us before we record the podcast.

Insights from listeners and audience data

To date, the podcast series has received around 2,400 downloads from listeners across 30 different countries. The audience survey was distributed by the Podcast team and received three responses (from a student radiographer, and two therapeutic radiographers). There is not enough data to draw any generalisable insights from the audience survey, however, we have summarised the findings below for reference:

- 100% of respondents agreed or strongly agreed that the podcast provided an accessible form of CPD.
- 100% of listeners tool insights from the patient's perspective, including lived experience, the importance of the patient's voice, and how research can inform the patient.
- 100% of the listeners learned something new related to the research that was shared on the podcast.

> *The importance of the patient's voice. So many considerations I had never had before. You think you are acting in the patient's best interests; however, not involving them means you never know if this is really the case. It was a lightbulb moment for me.*
>
> *One of the guests spoke about the importance of kindness and how patients aren't just people with tumours, they are mothers, fathers, husbands, wives, daughters and sons etc and this should always be kept in the forefront of your mind during treatment. As a student, it is really helpful listening to these podcasts to get the patient perspective so that we can aim to provide the best possible patient care when we enter the clinical environment.*
>
> *Live life and raise awareness for brain tumour research so it can receive the funding it deserves.*
>
> *How the patient voice must be considered and specifically the way I can improve practice to better support young people. I am already thinking of simple changes we could make in our department as a result of this work.*
>
> *(I learned about the…) variety of treatments available and the variances in the oncology pathway for young people. It was really interesting to hear about the reserach elements and how they helped inform the patient.*

- 100% of listeners felt that the podcast was a effective way to share research, and would listen for future podcasts. They each described different ways in which they listened to the podcast.

> *I found this a really useful way to access CPD and therefore would happily listen to other CPD based podcasts in order to stay up to date with knowledge and research*

You need to actively listen and so I couldn't multitask as I would if I was listening to a true crime podcast etc. However, it is a great way to listen to a topic and then prompt you to go on and do formalised CPD. Having the links to research and resources alongside the podcast episodes is great.

Easy way to learn whilst driving to work. I am a busy mum and so don't have time for formalised CPD outside of work and yet this was a great way for me to learn something and really reflect on my practice. I really looked at everything I did that day with a different lens on.

Conclusions and recommendations

This special podcast series provided a meaningful platform for cancer patients to share their experiences with peers, researchers, professionals and the wider public. Patient participants felt heard and welcomed as part of the process and felt like their contributions were valued. The interactions the patients had with researchers were viewed as being rewarding, both in terms of acquiring new knowledge about the research process and the people researching their condition, but also in providing meaning and a human context to the work that researchers do every day.

Researchers found the experience to be meaningful, and the interaction with patients was not only motivating but also insightful, particularly around the communication of science and the use of scientific terms. For some researchers, this was their first experience of patient or public engagement; for others, this was part of ongoing engagement. The programme was suitable for both experienced researchers and those with less experience.

The evidence we collected suggested that the level of support for participants was 'about right'. Potentially, there could be an opportunity for a dress rehearsal or role-play in the early part of the podcast in order to build confidence; equally, the programme team might consider a debrief with researchers to reflect on the experience and lessons learned. This de-brief could be facilitated with all participating researchers. To some extent, our evaluation, or future evaluations, might provide the opportunity for reflection and learning amongst peers.

The role of the programme facilitator was seen as essential in weaving together the different components of the programme and helping to develop the storyline. Given the potential challenges in matching participants with researchers, the fact that all participants felt well matched and the podcast conversations were coherent, this can be viewed a key success in the delivery of the programme.

The podcast team deliberately kept an informal feel to the podcasts and successfully provided space for both the patient and researcher experience. Whilst each podcast was different, the 'flow' of some podcasts felt more natural to the recording team. The feedback collected from the audiences suggests that… xx

In conclusion, the project delivered its stated aims. It provided a highly successful format for engaging researchers in informal conversations with cancer patients and gaining an understanding of their experiences; it also enabled researchers to develop interpersonal skills

and practice communicating their research to non-specialist audiences. Researchers gained some familiarity with the format of podcasts as a mechanism to communicate research but were also left feeling positive about patient and public involvement and engagement. Whilst it was not a stated aim, the fact that the experience also provided a much-needed channel for cancer patients to share their experiences and potentially inspire other cancer patients and professionals was vital to the success of the programme and key to its mutual benefit. The evidence also suggested that engaging cancer patients with research related to their disease was a valuable experience. The audience data suggested that the podcasts had been successful in increasing awareness of radiotherapy as an anti-cancer treatment and promoting UCL's cancer research to new and relevant audiences.

Recommendations

Based on our evaluation findings, we would put forward the following recommendations for development of this programme:

- Continued support of a dedicated PPIE coordinator.
- Consider a short preparation session, particularly for researchers or for patients and researchers together to build confidence.
- Consider offering a debriefing session with researchers to reflect on the process and the challenges of engaging patients and/or communicating their research to non-specialists.
- Where possible, encourage patients and researchers to find common ground beyond the cancer experience and research; for example, in one episode, the patient had also undertaken their own research.
- Set clear expectations to researchers, that the series is about them sharing themselves as much as it is about them sharing their research and knowledge. Provide models and examples of what this looks like in practice (e.g. sound clips from previous podcasts).
- Continue to utilise the format of podcasting conversations, as this provides a good conduit for patient engagement but also meets additional objectives around communicating research to broader audiences.
- Reflect on how audience insights were collected and identify opportunities to collect more audience data on future episodes.
- Consider the development of a case study to illustrate to internal staff, and the broader sector how podcasting can be used as both an engagement format, and communication tool.

Appendix A: List of podcast episodes

Jamie and Jesse - https://radchat.transistor.fm/episodes/bonus-episode-jesse-and-jaime-brain-tumours-survivorship-and-research

Michaela and Patycja - https://radchat.transistor.fm/episodes/bonus-episode

Elly and Amanda - https://radchat.transistor.fm/episodes/bonus-episode-elly-and-amanda-total-body-irradiation-and-patient-voice-in-research

Helen and Catarina - https://radchat.transistor.fm/episodes/bonus-episode-helen-haar-and-catarina-correia-velosa-da-veiga-non-hodgkin-s-lymphoma

Elena and Gemma - https://radchat.transistor.fm/episodes/bonus-episode-elena-espinosa-cabrera-and-dr-gemma-eminowicz-germ-cell-cancer

Sophie and Becca - https://radchat.transistor.fm/episodes/bonus-episode-sophie-lambert-and-rebecca-drake-non-hodgkin-s-lymphoma
